# Molecular mechanisms and crop improvement potential of RNA N^6^-methyladenosine in plants

**DOI:** 10.1007/s42994-025-00228-1

**Published:** 2025-08-04

**Authors:** Diyi Fu, Huiyuan Wang, Bochen Jiang

**Affiliations:** 1 School of Life Sciences & Biotechnology, Shanghai Jiao Tong University, Shanghai, 200240 China; 2 Sycamore Research Institute of Life Sciences, Shanghai, 201203 China

**Keywords:** RNA epigenetic, m^6^A, RNA metabolic, RNA regulon, Crop improvement

## Abstract

N^6^-methyladenosine (m^6^A) is the most prevalent internal modification in eukaryotic mRNAs and contributes to the post-transcriptional regulation of gene expression. In plants, m^6^A modulates RNA splicing, stability, and translation, thereby influencing developmental processes and responses to environmental stimuli. This review systematically examines current advances in the understanding of m^6^A regulation in plants. We begin with an overview of the m^6^A modification and its associated regulatory machinery, including the writers (methyltransferases), erasers (demethylases), and readers (m^6^A-binding proteins) components, and discuss their roles in orchestrating RNA metabolism and determining plant phenotypes. Subsequent sections focus on the functional implications of m^6^A in economically important crops, with evidence drawn from model systems such as *Arabidopsis thaliana* and key species including rice (*Oryza sativa*), tomato (*Solanum lycopersicum*), and strawberry (*Fragaria vesca*), where m^6^A modifications have been linked to traits such as yield, maturation, and aroma. Finally, we explore emerging biotechnological strategies that harness m^6^A-mediated regulatory pathways to enhance crop quality, such as overexpression of human *FTO* encoding an m^6^A demethylase, quantitative m^6^A profiling at single-base resolution, CRISPR/Cas13-targeted m^6^A regulation, the application of small-molecule inhibitors, and m^6^A-driven multi-omics integration. These strategies provide a comprehensive framework for understanding the multifaceted roles of m^6^A in plant biology and underscore the potential of this modification as a target for next-generation crop improvement.

## Introduction

**Fig. 1:**
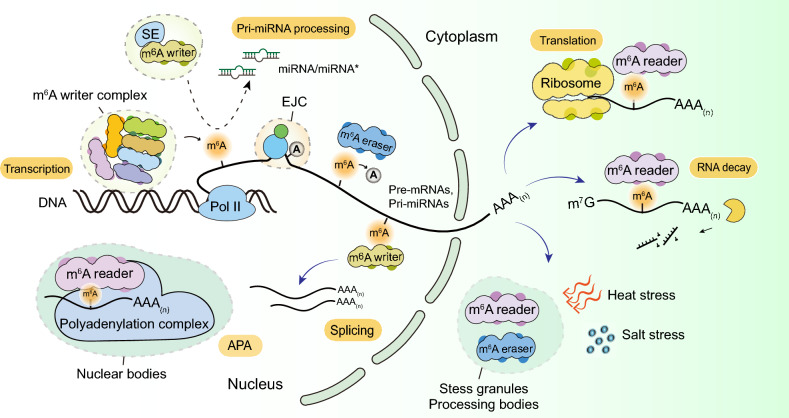
Molecular mechanisms of RNA m^6^A modification in plants. m^6^A “writers” (or a writer complex) catalyze the methylation of adenosine residues within RNA, “erasers” catalyze RNA demethylation, and “readers” recognize m^6^A-modified RNAs. Together, these components play essential roles in RNA metabolism by regulating transcription, polyadenylation, splicing, primary miRNA processing, translation, and RNA degradation

N^6^-methyladenosine (m^6^A) is widely recognized as the most abundant internal modification of eukaryotic messenger RNA (mRNA), serving as a crucial regulator of post-transcriptional gene expression (Roundtree et al. 2017). For decades, RNA has largely been viewed as the passive intermediate between DNA and protein synthesis; however, the discovery of m^6^A and other RNA modifications has revolutionized our understanding of RNA biology (Dominissini et al. 2012; Jia et al. 2011; Meyer et al. 2012). These modifications add an additional layer of regulation, allowing cells to fine-tune gene expression rapidly and dynamically in response to developmental cues and environmental stimuli (He and He 2021; Roundtree et al. 2017; Sendinc and Shi 2023). In plants, m^6^A methylation has emerged as a pivotal mechanism underlying various aspects of RNA metabolism, including transcription, polyadenylation, splicing, processing of primary microRNA (pri-miRNA), translation, and RNA degradation (Anderson et al. 2018; Duan et al. 2017; Luo et al. 2014; Shen et al. 2016; Sun et al. 2022; Wang et al. 2022; Wei et al. 2018) (Fig. 1). The reversible nature of this modification, controlled by the coordinated actions of methyltransferases (writers), demethylases (erasers), and m^6^A-binding proteins (readers), confers plants with the plasticity to rapidly adjust their transcriptomic landscape in response to internal developmental signals and external environmental changes (Tayier et al. 2024). For example, dynamic changes in m^6^A levels have been linked to developmental transitions such as flowering and embryogenesis, as well as responses to biotic and abiotic stresses (Tang et al. 2023).

The m^6^A regulatory machinery in plants is composed of three primary groups of proteins. In plants, mRNA m^6^A methylation is facilitated by two types of evolutionarily conserved RNA methyltransferases, methyltransferase A (MTA) and MTB, and FIONA1 (FIO1), identified as the functional analogs of Methyltransferase-like 3 (METTL3), METTL14, and METTL16, respectively, in human (*Homo sapiens*) (Cai et al. 2023; Růžička et al. 2017; Shen et al. 2016; Sun et al. 2022; Wang et al. 2022; Xu et al. 2022; Zhong et al. 2008). These enzymes work in concert with accessory proteins to ensure that m^6^A marks are installed at specific sites along a given transcript. By contrast, erasers such as the demethylase ALKBH10B remove this modification, thereby providing a mechanism for the dynamic modulation of m^6^A levels (Duan et al. 2017). Complementing this list of enzymes are m^6^A readers, predominantly members of the YT521-B homology (YTH) domain-containing protein family, which recognize and bind to m^6^A-modified transcripts, subsequently influencing RNA fate through various downstream actions including mRNA degradation and translational regulation (Amara et al. 2024; Arribas-Hernández et al. 2018, 2020, 2021b; Scutenaire et al. 2018; Wang et al. 2023a; Wei et al. 2018; Wu et al. 2024).

The dynamic interplay among writers, erasers, and readers ensures that the presence of m^6^A modifications is precisely regulated, allowing plants to adjust quickly to developmental signals and environmental challenges. Furthermore, emerging biotechnological strategies are beginning to exploit the dynamic nature of m^6^A regulation for crop improvement. Tools such as clustered regularly interspaced short palindromic repeats (CRISPR)/CRISPR-associated nuclease 13 (Cas13)-mediated targeted m^6^A regulation (Yu et al. 2024), quantitative profiling of m^6^A at single-base resolution (Wang et al. 2024a), and the modulation of m^6^A levels through overexpression of demethylase genes like human *Fat mass and obesity-associated protein* (*FTO*) are opening new avenues for fine-tuning gene expression (Yu et al. 2021). In addition, studies exploring m^6^A condensates (Jiang 2024; Shen 2024) and the application of small-molecule inhibitors (Deng et al. 2023) will accelerate our understanding of m^6^A-mediated regulatory networks and their potential for enhancing crop resilience and productivity. Thus, this review aims to systematically examine the recent advances in our understanding of m^6^A regulation in plants. By synthesizing findings from both model systems such as Arabidopsis (*Arabidopsis thaliana*) and key crop species such as rice (*Oryza sativa*), tomato (*Solanum lycopersicum*), and soybean (*Glycine max*), we provide a comprehensive framework that highlights the multifaceted roles of the m^6^A modification in plant development and its potential as a target for next-generation crop improvement.

## Overview of the m^6^A modification in plants

Understanding the m^6^A modification in plants requires an integrated examination of its chemical characteristics, detection methods, regulatory components, and functional consequences on RNA metabolism. In this section, we provide an in-depth discussion of these aspects, laying the groundwork for a subsequent exploration of how m^6^A influences plant phenotypes. 

### Detection and distribution

Accurate mapping and quantification of m^6^A modifications are essential for elucidating their functional roles. Measuring m^6^A levels in RNA initially employed methods such as thin-layer chromatography (TLC), dot-blot assays, and liquid chromatography–tandem mass spectrometry (LC–MS/MS). These approaches effectively assess overall m^6^A modification levels, allowing comparisons between wild-type plants and mutants deficient in specific m^6^A-related components (Tang et al. 2023). The subsequent advent of high-throughput sequencing methods such as m^6^A-seq or MeRIP-seq (methylated RNA immunoprecipitation sequencing) has since transformed our ability to detect m^6^A at global and single-transcript scales. These approaches rely on m^6^A-specific antibodies to enrich an extract in m^6^A-containing RNA fragments, which are then sequenced to reveal the m^6^A-enriched regions in specific transcripts (Dominissini et al. 2012; Meyer et al. 2012). Although powerful, these methods traditionally offer a resolution of about 100–200 bases, leaving ambiguity regarding the exact modification site(s).

Still using an anti-m^6^A antibody, advanced techniques such as miCLIP (m^6^A individual nucleotide resolution crosslinking and immunoprecipitation) (Linder et al. 2015) and m^6^A-LAIC-seq (m^6^A-level and isoform-characterization sequencing) (Molinie et al. 2016) were developed to enhance m^6^A detection. miCLIP and m^6^A-LAIC-seq achieve higher accuracy than m^6^A-seq and MeRIP-seq. Nevertheless, these antibody-dependent methods fail to achieve true single-base resolution, cannot accurately determine m^6^A stoichiometry, and do not possess the sensitivity required to effectively monitor dynamic changes in m^6^A methylation. Recently, single-base resolution methods such as m^6^A-SAC-seq (m^6^A-selective allyl chemical labeling and sequencing) (Hu et al. 2022), eTAM-seq (evolved TadA-assisted N^6^-methyladenosine sequencing) (Xiao et al. 2023), CAM-seq (chemical cooperative catalysis-assisted m^6^A sequencing) (Wang et al. 2025), and GLORI (developed glyoxal and nitrite-mediated deamination of unmethylated adenosines) (Liu et al. 2023; Zhou et al. 2025) have revealed extensive m^6^A dynamics by providing detailed information on site distribution and modification stoichiometry. These methods use specific enzymes or chemical reagents to modify the state of m^6^A or non-m^6^A sites, which can then be detected by sequencing. For example, SAC-seq employs a specific enzyme that attaches an allyl group to m^6^A, resulting in the formation of a^6^m^6^A, which then undergoes cyclization upon treatment with iodine (I_2_). During reverse transcription, the cyclized a^6^m^6^A causes the introduction of a mutation by the reverse transcriptase, which can be detected by sequencing. In plants, quantitative profiling of m^6^A using m^6^A-SAC-seq across the life cycles of rice and Arabidopsis has generated comprehensive resources that link single-base-resolution m^6^A sites in specific transcripts with putative functions (Wang et al. 2024a).

Advances in transcriptome-wide m^6^A mapping and sequencing technologies have enabled the identification of m^6^A sites, which are primarily enriched around the stop codon and within the 3′ untranslated regions (3′ UTRs) (Tang et al. 2023). These sites largely share the dominant m^6^A consensus motif RRACH (R = A or G; H = A or C or U) as well as other motifs such as URUAY (Y = C or U) or UGUAW (W = U or A) (Hu et al. 2024; Tang et al. 2023; Wang et al. 2024a). The patterns and motifs of m^6^A deposition are highly conserved among plants (such as Arabidopsis and rice), yeast (*Saccharomyces cerevisiae*), and mammals, suggesting that the machinery responsible for dynamically installing, removing, and interpreting m^6^A modifications is conserved across kingdoms. However, since the expression, activity, and interaction dynamics of m^6^A writers, erasers, and readers can be modulated by various environmental stimuli, this regulatory mechanism may enable sequence-specific RNA-binding proteins to recognize distinct motifs and thereby target particular RNAs in response to environmental cues. Thus, whether additional conserved motifs exist remains to be fully elucidated, and further studies combined with more advanced sequencing technologies may resolve these uncertainties.

### The m^6^A regulatory machinery

The reversible deposition of m^6^A marks on RNA is orchestrated by a suite of proteins broadly categorized into writers and erasers. Together, these proteins mediate the installation and removal of m^6^A modifications, while readers are responsible for decoding and interpreting these marks, thereby controlling the fate of mRNAs in a highly dynamic manner.

#### Writers (methyltransferases)

The m^6^A writers catalyze the methylation of adenosine residues within RNA. In Arabidopsis, the core methyltransferase complex closely resembles that in animals and comprises key components that include homologs of METTL3 and METTL14, known as MTA and MTB, respectively, along with several accessory subunits such as FKBP12-INTERACTING PROTEIN 37 (FIP37), VIRILIZER (VIR), HAKAI, and HAKAI-INTERACTING ZINC FINGER PROTEIN 2 (HIZ2) (Růžička et al. 2017; Shen et al. 2016; Zhang et al. 2022a; Zhong et al. 2008) (Table 1). In this complex, MTA provides the catalytic function while MTB serves as a cofactor that stabilizes the complex and enhances substrate specificity. Regulatory subunits such as FIP37, VIR, and HAKAI modulate the activity and subcellular localization of the writer complex, ensuring that m^6^A deposition is executed in a temporally and spatially controlled manner within the cell (Tayier et al. 2024). In addition to the MTA–MTB complex, FIO1, a homolog of human METTL16, acts as a methyltransferase targeting U6 small nuclear RNAs (snRNAs) and mRNAs (Jiang et al. 2023; Parker et al. 2022; Sun et al. 2022; Wang et al. 2022; Xu et al. 2022).

**Table 1:** List of m^6^A regulatory components and their functional roles in plants

Species	Gene name	Components	Function in plants	References
Arabidopsis	*MTA*, *FIP37*	Writer complex	Embryo development	Zhong et al. (2008)
	*MTA*	Writer	miRNA biogenesis	Bhat et al. (2020); Zhong et al. (2024)
	*MTA*	Writer	Plant immunity	Prall et al. (2023)
	*FIP37*	Writer complex	Shoot stem cell fate	Shen et al. (2016)
	*FIP37*	Writer complex	Auxin signaling pathway	Li et al. (2024)
	*MTA*, *MTB*, *FIP37*	Writer complex	Circadian clock	Wang et al. (2021)
	*MTA*, *MTB*, *FIP37*, *VIR*, *HAKAI*	Writer complex	Pleiotropic phenotypes	Růžička et al. (2017)
	*MTA*, *MTB*, *FIP37*, *VIR*, *HAKAI*	Writer complex	Cold stress response	Govindan et al. (2022); Vicente et al. (2023); Wang et al. (2023b)
	*MTA*, *MTB*, *VIR*, *HAKAI*	Writer complex	Salt stress tolerance	Hu et al. (2021)
	*MTA*, *VIR*	Writer complex	Dark-induced leaf senescence	Sheikh et al. (2024)
	*VIR*	Writer complex	Photosynthesis during photodamage	Zhang et al. (2022b)
	*HIZ2*, *HAKAI*	Writer complex	Auxin responses and root development	Zhang et al. (2022a)
	*FIO1*	Writer	Alternative splicing	Parker et al. (2022)
	*FIO1*	Writer	Flowering	Sun et al. (2022)
	*FIO1*	Writer	Photomorphogenesis and flowering	Wang et al. (2022)
	*FIO1*	Writer	Floral transition	Xu et al. (2022); Cai et al. (2023)
	*FIO1*	Writer	Chlorophyll homeostasis	Jiang et al. (2023)
	*FIO1*	Writer	Salt stress	Cai et al. (2024b)
	*ALKBH8B*	Eraser	Salt stress and abscisic acid responses	Huong et al. (2022)
	*ALKBH9B*	Eraser	Vascular movement of alfalfa mosaic virus	Martinez-Perez et al. (2021)
	*ALKBH9B*	Eraser	Abscisic acid response	Tang et al. (2022)
	*ALKBH9B*	Eraser	Heat stress response	Fan et al. (2023)
	*ALKBH9C*	Eraser	Abiotic stresses and abscisic acid response	Amara et al. (2022)
	*ALKBH10B*	Eraser	Floral transition	Duan et al. (2017)
	*ALKBH10B*	Eraser	Salt stress and abscisic acid responses	Shoaib et al. (2021); Tang et al. (2021)
	*ALKBH10B*	Eraser	Plant immunity	Prall et al. (2023)
	*CPSF30-L*	Reader	Nitrate signaling	Hou et al. (2021)
	*CPSF30-L*	Reader	Floral transition and abscisic acid response	Song et al. (2021)
	*CPSF30-L*	Reader	TuMV infection	Wei et al. (2024)
	*ECT1*	Reader	Salicylic acid-dependent stress responses	Lee et al. (2024)
	*ECT1*, *ECT9*	Reader	Plant immunity	Wang et al. (2023a)
	*ECT2*	Reader	Normal trichome branching	Wei et al. (2018); Scutenaire et al. (2018)
	*ECT2*, *ECT3*, *ECT4*	Reader	Developmental timing and morphogenesis	Arribas-Hernández et al. (2018)
	*ECT2*, *ECT3*, *ECT4*	Reader	Cell proliferation during plant organogenesis	Arribas-Hernandez et al. (2020)
	*ECT2*, *ECT3*, *ECT4*	Reader	Abscisic acid response	Song et al. (2023a)
	*ECT2*, *ECT3*, *ECT4*	Reader	Pattern-triggered immunity	Chen et al. (2024)
	*ECT8*	Reader	Abscisic acid perception	Wu et al. (2024)
	*ECT8*	Reader	Abiotic stress sensor	Cai et al. (2024a)
	*ECT12*	Reader	Abiotic stress responses	Amara et al. (2024)
	*FLK*	Reader	Floral transition	Amara et al. (2023)
Rice	*OsMTA2*, *OsFIP (OsFIP37)*	Writer complex	Early sporogenesis	Zhang et al. (2019)
	*OsMTA2*, *OseIF3h*	Writer complex	Pollen development	Huang et al. (2021)
	*OsEDM2L*	m^6^A-writer like	Tapetal degradation	Ma et al. (2021)
	*OsFIP37*	Writer complex	Male meiosis development	Cheng et al. (2022)
	*OsFIP37*	Writer complex	Rice microsporogenesis	Cheng et al. (2025)
	*OsALKBH5 (OsALKBH9)*	Eraser	Meiosis development	Xue et al. (2024)
	*OsALKBH9 (OsALKBH5)*	Eraser	Pollen development	Tang et al. (2024b)
	*OsYTH07*	Reader	Flowering	Cui et al. (2024)
Soybean	*GmMTA*s	Writer	Low-blue-light response	Zhang et al. (2024a)
	*GmMTA2*, *GmMTB1*	Writer	Abiotic stress response	Liu et al. (2024a)
	*GmALKBH10B*s	Eraser	Abiotic stress response	Zhao et al. (2024b)
Wheat	*TdFIP37*	Writer	Salt stress response	Huang et al. (2024)
	*TaETC9*	Reader	Drought response	Pan et al. (2024)
Maize	*ZmMTA*	Writer	Kernel development	Luo et al. (2024)
Sorghum	*SbMTA*	Writer	Salt tolerance	Zheng et al. (2024)
	*SbALKBH10B*	Eraser	Salt tolerance	Zheng et al. (2024)
Foxtail millet	*SiYTH1*	Writer	Drought tolerance	Luo et al. (2023)
Tomato	*SlMTA*, *SlHAKAI*	Writer	PepMV infection	He et al. (2023a, 2023c)
	*SlMTC*	Writer	Salt stress	Gao et al. (2024)
	*SlALKBH2*	Eraser	Fruit ripening	Zhou et al. (2019)
	*SlALKBH2*	Eraser	Fruit ripening	Zhou et al. (2025)
	*SlALKBH2*	Eraser	DNA damage response	Tan et al. (2022)
	*SlALKBH10B*	Eraser	Drought and salt tolerance	Shen et al. (2024)
	*SlYTH2*	Reader	Fruit aroma	Bian et al. (2024)
	*SlYTH1*	Reader	Fruit shape	Yin et al. (2022)
	*SlYTP8/9*	Reader	Low temperature and waterlogging stress	Zhang et al. (2024c)
Apple	*MdMTA*	Writer	Drought tolerance	Hou et al. (2022)
	*MdVIR1/2*	Writer complex	Necrotrophic pathogen response	Song et al. (2025)
	*MhYTP2*	Reader	Powdery mildew resistance	Guo et al. (2022)
Strawberry	*FvMTA*, *FvMTB*	Writer	Fruit ripening	Zhou et al. (2021)
	*FvALKBH10B*	Eraser	Fruit ripening	Tang et al. (2024a)
Cotton	*GhALKBH5*	Eraser	Photoperiod sensitivity	He et al. (2024)
	*GhALKBH10*	Eraser	Salt tolerance	Cui et al. (2022)
	*GhALKBH10*	Eraser	Drought tolerance	Li et al. (2023)
*N. benthamiana*	*NbMTA*, *NbHAKAI*	Writer complex	PepMV infection	He et al. (2023a)
	*NbECT2A*, *NbECT2B*, *NbECT2C*	Reader	PepMV infection	He et al. (2023a)
Peanut	*AhALKBH15*	Eraser	Bacterial wilt response	Zhao et al. (2024a)
Kiwifruit	*AcALKBH10*	Eraser	Fruit ripening and quality	Su et al. (2024)
*Catalpa fargesii*	*CfALKBH5*	Eraser	Pigment accumulation	Zhang et al. (2023)
Switchgrass	*PvALKBH10*	Eraser	Flowering time	Liu et al. (2024b)
*Populus trichocarpa*	*PtrMTA*, *PtrFIP37*	Writer	Drought tolerance	Lu et al. (2020)
	*PagALKBH9B*,* PagALKBH10B*	Eraser	Salt stress response	Zhao et al. (2022)

Beyond Arabidopsis, METTL3/14-type methyltransferase complexes have been identified in at least ten plant species, including rice, soybean, and tomato. For instance, in rice, OsFIP (also reported as OsFIP37) and OsMTA2 interact to regulate early microspore degeneration via NTPase genes (*LOC_Os02g11050* and *LOC_Os02g10640*) (Zhang et al. 2019); in tomato, SlMTA and SlHAKAI inhibit infection by the pepino mosaic virus (PepMV) through m^6^A modification of the viral RNA (He et al. 2023a, 2023c). In soybean, GmMTAs modulate the shade avoidance response by altering the expression of genes whose transcripts are m^6^A-methylated (Zhang et al. 2024b). Despite these advances, the functional roles of METTL16-type methyltransferases in other plant species are largely unexplored.

#### Erasers (demethylases)

Counteracting the activity of m^6^A writers, m^6^A erasers remove methyl groups from m^6^A-modified RNAs, thus reversing the modification and allowing for dynamic regulation of gene expression. In mammals, the primary m^6^A demethylases include FTO and ALKBH5 (alkylated DNA repair protein AlkB homolog 5), both members of the Fe(II)/α-ketoglutarate-dependent dioxygenase superfamily (Jia et al. 2011; Zheng et al. 2013). In plants, several AlkB homologs have been identified across at least 12 species (see Table 1), although no clear homolog of FTO has been reported. In Arabidopsis, ALKBH8B, ALKBH9B, ALKBH9C, and ALKBH10B have been confirmed or proposed as m^6^A demethylases (Duan et al. 2017; Huong et al. 2022; Martínez-Pérez et al. 2017, 2021; Tang et al. 2022). Among these, ALKBH10B is the most thoroughly characterized; it plays a significant role in modulating m^6^A levels in mRNA, without affecting the methylation of transfer RNA (tRNA) (Duan et al. 2017). While other members of the AlkB family may also contribute to m^6^A demethylation, their specific roles and substrate preferences are unknown.

Notably, heterologous expression of human *FTO* in rice and potato (*Solanum tuberosum*) has been reported to significantly enhance yield by modulating m^6^A-regulated gene expression (Yu et al. 2021). Moreover, studies across different plant species have revealed that AlkB homologs contribute to various stress responses in tomato, soybean, cotton (*Gossypium hirsutum*), and sorghum (*Sorghum bicolor*); influence fruit ripening and quality in tomato, strawberry (*Fragaria vesca*), and kiwifruit (*Actinidia chinensis*); and affect meiosis and pollen development in rice, as well as flowering in switchgrass (*Panicum virgatum*) (Cui et al. 2022, 2024; Shen et al. 2024; Su et al. 2024; Tang et al. 2024b; Xue et al. 2024; Zhou et al. 2019, 2021, 2025). These findings suggest that the strategic regulation of demethylases in plants may offer a promising approach for raising crop yields and improving stress tolerance. However, further research is needed to elucidate the precise underlying mechanisms and to develop effective strategies for their practical application.

#### Readers (m^6^A-binding proteins)

The functional outcome of m^6^A modification is largely determined by m^6^A readers, which specifically recognize and bind to methylated RNA. Upon binding to m^6^A sites, these proteins recruit additional effector proteins or complexes that subsequently modulate mRNA fate. To date, various m^6^A recognition proteins have been identified in mammals, including members of the YTH domain–containing family, insulin-like growth factor 2 mRNA-binding proteins (IGF2BPs), HNRNPA2B1, eukaryotic initiation factor 3 (eIF3), and heterogeneous nuclear ribonucleoprotein C (HNRNPC) (He and He 2021; Roundtree et al. 2017; Sendinc and Shi 2023; Tang et al. 2023). In Arabidopsis, the most extensively studied m^6^A readers belong to the YTH domain–containing protein family. This group includes EVOLUTIONARILY CONSERVED C-TERMINAL REGION 1 (ECT1), ECT2, ECT3, ECT4, ECT8, ECT9, ECT12, and the long isoform of CLEAVAGE AND POLYADENYLATION SPECIFICITY FACTOR 30 (CPSF30-L), all of which possess a conserved YTH domain through which they bind to m^6^A-modified nucleotides (Table 1). Through this binding, they influence polyadenylation, mRNA stability, and translation (Amara z et al. 2024; Arribas-Hernandez et al. 2020, 2021a, 2021b; Cai et al. 2024b; Hou et al. 2021; Lee et al. 2024; Scutenaire et al. 2018; Song et al. 2021, 2023b; Wang et al. 2023a; Wei et al. 2018; Wu et al. 2020). Notably, a recent study identified FLOWERING LOCUS K (FLK), a K homology domain protein, as a previously unknown mRNA m^6^A reader that regulates the floral transition in Arabidopsis (Amara et al. 2023). In other plant species, m^6^A readers also contribute significantly to the regulation of development and stress responses. For example, in rice, EARLY HEADING DATE 6 (EHD6) recruits the m^6^A reader YTH07, sequestering *CONSTANS-LIKE 4* (*OsCOL4*) mRNA into phase-separated ribonucleoprotein condensates to promote flowering (Cui et al. 2024). In tomato, SlYTH2 represses the translation of target m^6^A-modified mRNAs through its phase separation ability (Bian et al. 2024). Similarly, in foxtail millet (*Setaria italica*), SiYTH1 enhances drought tolerance by modulating the stability of mRNAs encoding proteins involved in stomatal closure and reactive oxygen species scavenging (Luo et al. 2023). Mutations or altered expression of the corresponding m^6^A reader genes frequently lead to pronounced phenotypic changes, underscoring the crucial role of m^6^A recognition in the regulation of gene expression.

## Functional implications of m^6^A in crops

**Fig. 2:**
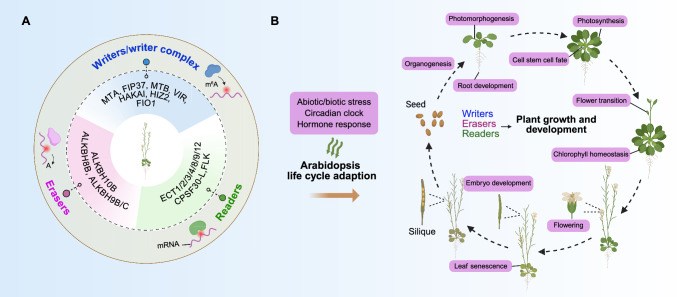
Biological functions of m^6^A in Arabidopsis. **A** Identified m^6^A writers, erasers, and readers in Arabidopsis. **B** Biological functions of the m^6^A modification in plant growth, development, and environmental adaptation, including flowering, photosynthesis, root development, chlorophyll homeostasis, organogenesis, leaf senescence, stem cell fate, phytohormone responses, and responses to abiotic or biotic stress

**Fig. 3:**
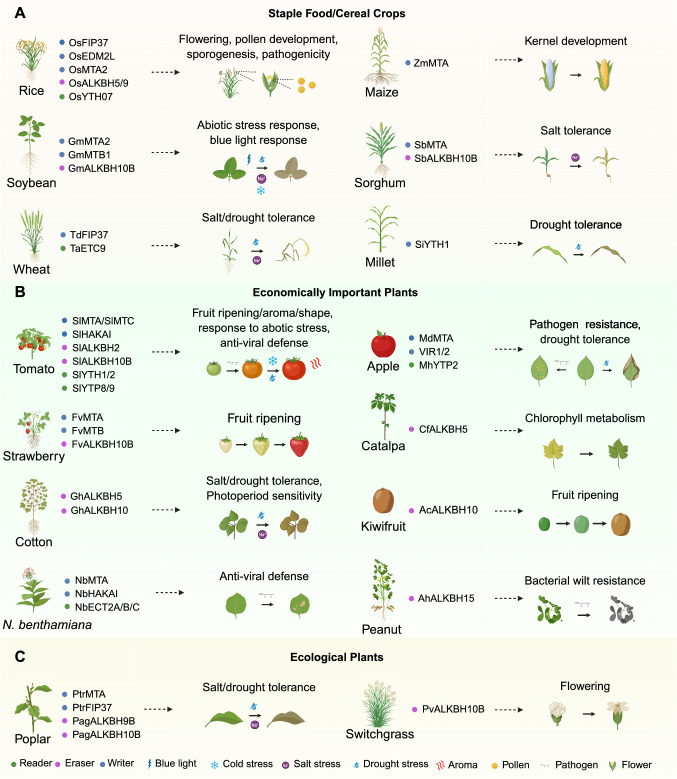
m^6^A components and their biological functions in crops. Identified m^6^A writers (annotated in blue), erasers (magenta), and readers (green) in staple food/cereal crops (**A**), economically important crops (**B**), and ecological plant species (**C**). Recent studies have shown that numerous m^6^A components in 16 different crops play roles in crop growth, development, and responses to environmental stimuli

The regulation of m^6^A deposition in plants involves a coordinated network of writers and erasers, which together with m^6^A readers control key aspects of RNA life such as transcription, polyadenylation, splicing, primary miRNA processing, translation, and RNA degradation (Fig. 1). The post-transcriptional regulation conferred by the m^6^A modification has garnered significant attention for its potential to influence a broad spectrum of agronomic traits in plants. Building upon insights from the model plant Arabidopsis, where m^6^A plays crucial roles in embryo development, shoot stem cell fate, circadian rhythms, and stress responses (Fig. 2), researchers have extended their investigations to a diverse array of crops. Detailed studies in staple food/cereal crops (Fig. 3A, including rice, soybean, wheat [*Triticum aestivum* L.], maize, foxtail millet, and sorghum), economically important plants (Fig. 3B, including tomato, apple [*Malus domestica*], strawberry, peanut [*Arachis hypogaea*], kiwifruit, *Catalpa fargesii*, cotton, and *Nicotiana benthamiana*), and ecological plants (Fig. 3C, including poplar [*Populus trichocarpa*] and switchgrass) demonstrate that the m^6^A modification of RNA affects yield, developmental timing, stress resilience, and quality traits (Fig. 3). In this section, we review evidence from model systems, present in-depth case studies across several key crop species, and provide a comparative analysis that highlights both conserved and species-specific roles of m^6^A modification.

### Insights from Arabidopsis studies

Arabidopsis has served as a fundamental model for elucidating the functional roles of the m^6^A modification in plants (Fig. 2). The writer complex has been shown to modulate essential developmental processes including embryo development, shoot stem cell fate, and circadian rhythms (Shen et al. 2016; Wang et al. 2021; Zhong et al. 2008). For example, inactivation of MTA results in embryonic arrest at the globular stage (Zhong et al. 2008), while mutants lacking FIP37 function exhibit excessive shoot meristem proliferation alongside a transcriptome-wide drop in m^6^A levels. Further investigations have demonstrated that FIP37 mediates the deposition of m^6^A on transcripts of critical shoot meristem regulator genes, such as *WUSCHEL* (*WUS*) and *SHOOT MERISTEMLESS* (*STM*), thereby promoting their degradation and maintaining appropriate transcript levels to prevent over proliferation (Shen et al. 2016). In addition to developmental regulation, writer components are pivotal in stress responses, contributing to cold tolerance, salinity stress adaptation, and dark-induced leaf senescence (Govindan et al. 2022; Hu et al. 2021; Sheikh et al. 2024; Vicente et al. 2023; Wang et al. 2023b). The demethylase ALKBH10B, for instance, affects the stability of its target transcripts; its loss delays flowering and represses vegetative growth, thus influencing the floral transition (Duan et al. 2017). Similarly, eraser proteins have been reported to modulate stress responses: ALKBH9B regulates abscisic acid (ABA) signaling and the heat stress response (Fan et al. 2023; Tang et al. 2022), while ALKBH8B is implicated in salinity stress tolerance (Huong et al. 2022). The regulatory network is further refined by m^6^A readers, particularly YTH domain-containing proteins such as ECT2, ECT3, ECT4, ECT8, and CPSF30-L. These readers govern a variety of programs, from trichome branching to mRNA stability under stress conditions. Notably, ECT2, ECT3, and ECT4 collectively enhance mRNA stability in response to ABA (Song et al. 2023b), whereas ECT8 forms phase-separated condensates that modulate ABA perception and function as an abiotic stress sensor by accelerating mRNA degradation (Cai et al. 2024b; Wu et al. 2024). The wide-ranging phenotypes associated with altered m^6^A dynamics in Arabidopsis establish a strong conceptual framework that has guided research in other plant species.

### Studies in staple food/cereal crops

#### Rice

Rice is one of the most extensively studied crops for m^6^A-related processes. The writer complex in rice mirrors that of Arabidopsis, with OsMTA2 and OsFIP37 playing crucial roles in early sporogenesis and pollen development (Cheng et al. 2025; Huang et al. 2021). Their interaction ensures appropriate m^6^A deposition on key transcripts; knocking out *OsFIP* results in early degeneration of microspores at the vacuolated pollen stage and simultaneously causes abnormal meiosis (Zhang et al. 2019). In addition, OsFIP37-ASSOCIATED PROTEIN 1 (OsFAP1) recruits OsFIP37 to mediate the m^6^A modification of transcripts for the auxin biosynthetic gene *OsYUCCA3* during male meiosis development (Cheng et al. 2022). Another methyltransferase-like factor, ENHANCED DOWNY MILDEW 2-LIKE (OsEDM2L), deposits m^6^A on the *EARLY ACTIVATION TAGGED 1* (*EAT1*) transcript, facilitating its proper alternative splicing and polyadenylation during tapetal cell death (Ma et al. 2021). Demethylases also figure prominently in rice development. OsALKBH5 (also reported as OsALKBH9) is required for normal meiosis, as it modulates mRNA stability for genes involved in double-strand break formation and repair (Xue et al. 2024). In addition, OsALKBH9-catalyzed m^6^A demethylation regulates tapetal programmed cell death (PCD) and pollen exine accumulation, both critical steps in ensuring male fertility (Tang et al. 2024a). On the reader side, YTH07 illustrates how m^6^A-binding proteins can fine-tune developmental processes: it interacts with EHD6 and sequesters *OsCOL4* mRNA in phase-separated condensates to promote flowering (Cui et al. 2024). Together, these findings highlight how m^6^A dynamics in rice influence a broad suite of developmental processes, spanning meiosis, pollen formation, and flowering, that directly affect yield. Understanding and manipulating these regulatory nodes may offer innovative avenues to boost productivity in one of the world’s most important cereal crops.

#### Soybean

Soybean is another major crop in which m^6^A plays pivotal roles in stress adaptation and development. Members of the writer complex, including GmMTAs, have been shown to regulate the shade avoidance response by altering gene expression through the m^6^A modification of *CRYPTOCHROME 1* (*GmCRY1*), *SUPPRESSOR OF PHYA-105* (*GmSPA*), and *CONSTITUTIVE PHOTOMORPHOGENIC 1* (*GmCOP1*) transcripts. In environments with low blue-light intensity or under canopy shade, these m^6^A-regulated genes influence plant architecture and resource allocation (Zhang et al. 2024b). Besides, overexpressing *GmMTA2* or *GmMTB1* in soybean alters plant tolerance to alkaline stress and long-term darkness, confirming their effects on stress responses (Liu et al. 2024b). Demethylases also contribute to soybean stress responses. The expression of *GmALKBH10B* genes responds differentially to various abiotic stresses, hinting at a nuanced regulatory landscape wherein each demethylase isoform may target distinct sets of transcripts (Zhao et al. 2024a). By fine-tuning gene expression in response to environmental stimuli, these demethylases can help soybean plants reach a balance between growth and defense, ultimately affecting yield and resilience. Although soybean research on m^6^A lags slightly behind that in Arabidopsis and rice, current findings strongly suggest that manipulating the m^6^A machinery could improve high-density planting potential and enhance plant tolerance under challenging environmental conditions.

#### Wheat

Wheat (*Triticum aestivum L.*) is another major cereal crop whose m^6^A-mediated regulatory mechanisms modulate responses to abiotic stress. Notably, the writer-associated factor TdFIP37 in wild emmer (*Triticum dicoccoides*) enhances salinity tolerance, primarily through its modulation of the mitogen-activated protein kinase (MAPK) signaling pathway, which is critical for activating stress-responsive gene networks (Huang et al. 2024). Moreover, functional studies have revealed that loss-of-function mutations in the m^6^A reader gene *TaECT9* lead to greater drought sensitivity, underscoring the essential role of m^6^A recognition in maintaining water-stress resilience (Pan et al. 2024). Together, these findings illustrate how precise m^6^A dynamics are integral to orchestrating the complex gene regulatory networks behind tolerance to diverse abiotic stresses, offering promising targets for improving stress resilience in wheat cultivars.

#### Maize

In maize (*Zea mays*), the writer ZmMTA is responsible for depositing m^6^A marks specifically on the 5′ UTRs of transcripts from key regulatory genes that control kernel formation. Notably, recent evidence indicates that ZmMTA facilitates m^6^A deposition on RNA and influences DNA methylation, such as the mCHH mark, in the corresponding genomic regions. The presence of m^6^A on the 5′ UTR of RNA, and possibly mCHH marks on their associated genes, is thought to modulate chromatin accessibility and transcriptional dynamics, thus influencing gene expression patterns critical for cell division, differentiation, and nutrient allocation during kernel development (Luo et al. 2024). As kernel size and composition are major determinants of maize yield, ZmMTA appears to have a pivotal role in fine-tuning the epitranscriptomic landscape. Variations in ZmMTA activity can lead to differential expression of developmental regulator genes, ultimately affecting kernel morphology and overall productivity. Thus, better understanding ZmMTA function and how to potentially manipulate it may offer new avenues for enhancing maize yield through targeted epitranscriptomic interventions.

#### Sorghum

In sorghum, m^6^A modifications mediate tolerance to salinity stress through phytohormonal regulation. The writer SbMTA deposits m^6^A marks on target transcripts, whereas the eraser SbALKBH10B actively removes these modifications (Zheng et al. 2024). The coordinated actions of SbMTA and SbALKBH10B modulate the overall m^6^A landscape, which in turn influences the ABA signaling pathway, a central mechanism in the salinity stress response. ABA initiates and coordinates plant responses to saline environments; thus, m^6^A regulation is essential for the appropriate expression of ABA-responsive genes. Enhanced or altered expression of *SbMTA* or *SbALKBH10B* has been correlated with adjustments in m^6^A levels on transcripts encoding key stress-responsive proteins, contributing cellular homeostasis and sustaining growth under salinity stress. This finely tuned epitranscriptomic regulation suggests that manipulating SbMTA and/or SbALKBH10B activity could be a promising strategy for improving salinity stress tolerance and, consequently, yield in sorghum cultivated in saline soils.

#### Foxtail millet

In foxtail millet, the m^6^A reader SiYTH1 is central to conferring drought tolerance by regulating the stability of mRNAs that encode proteins involved in critical drought response pathways. SiYTH1 specifically recognizes and binds to m^6^A-modified transcripts that govern stomatal closure and reactive oxygen species (ROS) scavenging. Efficient stomatal closure is essential for minimizing water loss during drought, while the timely removal of ROS prevents cellular damage under stress conditions. By modulating the stability of these mRNAs, SiYTH1 ensures that stress-responsive genes are expressed at optimal levels when water availability is limited. This post-transcriptional regulation is vital for cellular homeostasis, as it balances the need for water conservation with the metabolic demands of stress adaptation (Luo et al. 2023). The precise action of SiYTH1 in stabilizing key transcripts under drought conditions ultimately contributes to improved water-use efficiency and enhances the overall drought tolerance of foxtail millet, making it a resilient crop in arid environments.

### Studies in economically important plants

#### Tomato

Tomato serves as a prime example of how m^6^A regulation can affect fruit development and quality (Bian et al. 2024; Gao et al. 2024; He et al. 2023a; Shen et al. 2024; Tan et al. 2022; Yin et al. 2022; Zhang et al. 2024c, 2024d; Zhou et al. 2019, 2025). The writer complex, including SlMTA and SlHAKAI, has been implicated in restricting PepMV infection by increasing viral RNA m^6^A modifications and inducing its degradation via nonsense-mediated decay (NMD), underscoring m^6^A’s role in antiviral defense (He et al. 2023a). The demethylase SlALKBH2 influences fruit ripening by modulating the stability of *SlDML2* transcripts, encoding a DNA demethylase crucial for ripening progression (Zhou et al. 2019). Redox regulation of SlALKBH2, in turn, affects its protein stability, illustrating how multiple layers of regulation converge onto m^6^A deposition and interpretation (Zhou et al. 2025). Other members of the AlkB family also modulate stress tolerance and developmental programs in tomato. For instance, SlALKBH10B negatively regulates drought and salinity tolerance (Shen et al. 2024). YTH domain readers like SlYTH2 exert control over fruit aroma and quality by repressing the translation of target mRNAs via phase separation, a physical process allowing cells to compartmentalize biomolecules independent of a bounding membrane. This ability to partition specific transcripts into distinct subcellular compartments may be key to fine-tuning the biosynthesis of flavor-related compounds (Bian et al. 2024). In addition, SlYTH1 has been linked to fruit shape by modulating gibberellin biosynthesis (Yin et al. 2022). Taken together, studies in tomato highlight how m^6^A influences plant–pathogen interactions and abiotic stress responses as well as traits directly related to consumer preferences, such as flavor and aroma.

#### Strawberry

In strawberry, m^6^A modification can directly influence fruit quality attributes like aroma. The writer components FvMTA and FvMTB modulate the stability and translation efficiency of transcripts for key genes such as *NINE-CIS-EPOXYCAROTENOID DIOXYGENASE 5* (*NCED5*), *ABSCISIC ACID RESPONSIVE ELEMENTS-BINDING PROTEIN 1* (*AREB1*), and putative *ABA RECEPTOR* (*ABAR*), all of which are involved in ABA signaling pathways (Zhou et al. 2021). As ABA plays a critical role in strawberry fruit ripening, changes in m^6^A deposition can lead to alterations in ripening dynamics and flavor development. The demethylase FvALKBH10B controls fruit ripening through the FvABF3–FvALKBH10B–FvSEP3 cascade consisting of ABA-RESPONSIVE ELEMENT BINDING FACTOR 3 (FvABF3), FvALKBH10B, and SEPALLATA 3 (FvSEP3) (Tang et al. 2024b). This regulatory module points to a broader network wherein m^6^A marks can modulate phytohormonal cues and developmental genes to fine-tune ripening stages. By affecting aroma and overall fruit quality, m^6^A regulation in strawberry highlights the direct agricultural relevance of epitranscriptomic modifications for consumer-centric traits.

#### Apple

Apple offers another example as to how the m^6^A modification orchestrates development and stress responses, influencing fruit quality and yield. In apple, the writer complex includes MdMTA, which promotes m^6^A deposition to enhance the stability and translation efficiency of key mRNAs involved in lignin deposition and oxidative stress management (Hou et al. 2022), and accessory factors such as MdVIR1 and MdVIR2. These VIR proteins mediate resistance against necrotrophic pathogens; specifically, sorbitol regulates the expression of MdVIR1 and MdVIR2. The m^6^A modifications facilitated by these genes are essential for sorbitol-controlled resistance to the fungus *Alternaria alternata*. Thus, MdVIR1 and MdVIR2 link metabolic signals with immune responses in apple (Song et al. 2025). On the reader side, MhYTP2 from the Chinese crabapple (*Malus hupehensis*) reinforces these defense mechanisms by elevating m^6^A modification levels and enhancing the translation of defense-related transcripts, thereby increasing resistance to powdery mildew (Guo et al. 2022). Together, the coordinated actions of MdMTA, MdVIR1, MdVIR2, and MhYTP2 demonstrate that m^6^A modifications in apple support cellular homeostasis and growth under stress conditions while directly contributing to improved pathogen resistance and fruit quality, underscoring the potential of targeting epitranscriptomic regulation for crop improvement in this crop species.

#### Other economic crops

In addition to tomato, strawberry, and apple, several other economically important plant species employ m^6^A‐mediated regulatory mechanisms to influence stress responses, development, and quality traits. For instance, in cotton, the m^6^A eraser GhALKBH10 enhances salinity and drought tolerance by raising antioxidant capacity and lowering cytoplasmic sodium (Na^+^) levels, thus helping to support sustained growth under adverse conditions (Cui et al. 2022; He et al. 2024; Li et al. 2023). In *Nicotiana benthamiana* (*N. benthamiana*), a relative of tobacco (*Nicotiana tabacum*), the coordinated action of the writer components NbMTA and NbHAKAI, together with m^6^A readers such as NbECT2A, NbECT2B, and NbECT2C, plays a key role in antiviral defense against PeMV by facilitating the degradation of viral RNAs and stabilizing defense-related transcripts (He et al. 2023a). Similarly, in peanut, the m^6^A eraser AhALKBH15 is essential for bacterial wilt resistance through dynamic modulation of m^6^A levels on the transcripts of key defense genes (Zhao et al. 2024b). In kiwifruit, m^6^A regulation is particularly crucial for fruit development and quality; the demethylase AcALKBH10 modulates the removal of m^6^A marks on transcripts associated with ripening, thereby affecting mRNA stability and translation efficiency. This regulation influences the timing and progression of fruit maturation, ultimately influencing fruit texture, flavor, and overall marketability (Su et al. 2024). Moreover, in Maiyuanjinqiu, a natural variety cultivated from the seedings of the Chinese bean tree (*Catalpa fargesii*), the m^6^A demethylase CfALKBH5 is critical for proper pigment accumulation; the silencing of its encoding gene results in a pronounced chlorotic phenotype, likely due to impaired expression of genes involved in chlorophyll biosynthesis and the maintenance of chloroplast function (Zhang et al. 2023). Collectively, these findings underscore the diverse yet integral roles of m^6^A modifications across a range of economically important crops, highlighting the potential of targeting epitranscriptomic regulatory pathways to enhance stress resilience, improve disease resistance, and optimize quality traits in agricultural systems.

### Studies in ecological plants

In switchgrass, the m^6^A eraser PvALKBH10 dynamically modulates the m^6^A levels of transcripts that govern developmental timing, particularly the transition to flowering. By precisely controlling the stability and translation of these key regulatory mRNAs, PvALKBH10 ensures that flowering is optimally timed to coincide with favorable environmental conditions, thereby maximizing biomass production and enhancing switchgrass adaptability to fluctuating climates (Liu et al. 2024a).

Similarly, in popla, a woody perennial of significant ecological and economic importance, the writer components PtrMTA and PtrFIP37 are instrumental for enhancing drought tolerance. Indeed, they deposit m^6^A marks on transcripts that regulate the development of trichomes and roots, the structures essential for efficient water uptake and retention (Lu et al. 2020). Concurrently, the demethylases PagALKBH9B and PagALKBH10B, identified in the poplar hybrid genotype *P. alba* × *P. tremula* var. *glandulosa*, fine-tune m^6^A removal, ensuring that gene expression is dynamically adjusted in response to salinity stress (Zhao et al. 2022). The coordinated actions of these m^6^A regulators underscore a sophisticated mechanism that balances growth with stress adaptation, helping poplar in maintaining robust development and survival under adverse environmental conditions.

### Comparative analysis of m^6^A regulation across plant species

Functional studies of the m^6^A modification landscape across diverse crop species have revealed striking similarities in signaling pathways and regulatory mechanisms, suggesting conserved roles for the m^6^A machinery in plant development and stress responses. For instance, writer complex components such as MTA and FIP37 consistently mediate critical stages of development across Arabidopsis, rice, maize, and soybean, highlighting a common regulatory framework governing embryo development, reproductive organ formation, and circadian rhythms. Similarly, the demethylases ALKBH9 and ALKBH10 have been repeatedly shown to modulate stress responses, particularly to drought, salinity, and heat, in staple crops including rice, soybean, wheat, maize, sorghum, and foxtail millet. In addition, YTH domain-containing m^6^A reader proteins exhibit conserved roles in ABA signaling and the regulation of mRNA stability under abiotic stress conditions (Table 1). These commonalities underscore an evolutionarily conserved network integrating m^6^A regulation with key signaling pathways, thereby suggesting that insights gained from model systems like Arabidopsis can be leveraged to enhance stress resilience and optimize developmental traits across a wide array of crop species.

## Targeting m^6^A for crop quality enhancement

**Fig. 4:**
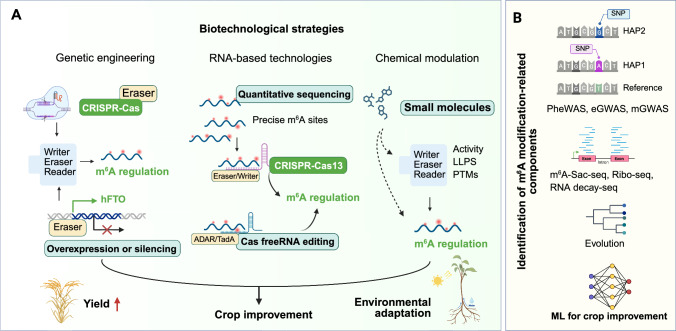
Biotechnological strategies to enhance crop quality. **A** Potential epigenetic control strategy, such as genetic engineering-mediated modification of m^6^A components, quantitative profiling-guided RNA editing of m^6^A-modified mRNA, and small molecule-mediated m^6^A regulation, may help enhance crop yield and plant adaptation. **B** m^6^A-driven multi-omics integration with machine learning (ML). High-throughput techniques can be used to identify components related to m^6^A modification

Building on the comprehensive understanding of how the m^6^A modification regulates plant development, stress adaptation (Figs. 2 and 3), and quality traits, emerging biotechnological strategies are now being explored to harness these epitranscriptomic mechanisms for crop improvement. These innovative approaches aim to manipulate m^6^A levels and dynamics with high precision, offering promising avenues for enhancing yield, stress resilience, and quality attributes in economically relevant crops. Here, we discuss several strategies, including genetic engineering-mediated modification of m^6^A components, quantitative profiling-guided RNA editing of m^6^A-modified mRNA, small molecule-mediated m^6^A regulation, and m^6^A-driven multi-omics integration with machine learning (ML), each with its underlying mechanism and potential applications (Fig. 4).

### Genetic engineering-mediated modification of m^6^A components

Genetic engineering provides a direct avenue for modulating the abundance of m^6^A regulatory proteins, thereby reprogramming the epitranscriptomic landscape in crops. This approach involves either overexpressing or silencing key genes of the m^6^A machinery, including those encoding the writers, erasers, and readers, to achieve precise control over m^6^A deposition. Recent research has demonstrated that heterologous expression of the human demethylase gene *FTO* in rice and potato enhances yields and improves stress tolerance. FTO mediates extensive m^6^A demethylation of plant RNAs in these plants, which is associated with greater chromatin accessibility and transcriptional activation. Moreover, heterologous expression of *FTO* stimulates the proliferation of root meristematic cells, promotes tiller bud formation, enhances photosynthetic efficiency, and improves drought tolerance in rice (Yu et al. 2021). These findings underscore the promise of modulating RNA m^6^A methylation as a strategy for enhancing plant growth and crop yield. Further investigation into the functions of m^6^A components using techniques such as T-DNA insertions, ethyl methanesulfonate (EMS)-induced chemical mutagenesis, and CRISPR/Cas-based genome editing will further elucidate their roles in determining crop quality.

### Quantitative profiling-guided RNA editing of m^6^A-modified mRNA

Advances in detection techniques have revolutionized our ability to map m^6^A modifications with unprecedented precision. Recent methodologies, such as m^6^A-SAC-seq, eTAM-seq, and GLORI, enable single-base-resolution profiling of m^6^A marks across the transcriptome (Hu et al. 2022; Liu et al. 2023; Xiao et al. 2023). These methods overcome the limitations of earlier antibody-dependent approaches by providing exact modification sites, accurate stoichiometry, and dynamic temporal resolution. These advances will help precisely correlate specific m^6^A sites with functional outcomes in the regulation of gene expression. In crop species, such high-resolution mapping will facilitate the identification of critical m^6^A modification sites that control yield, stress responses, and quality traits. In turn, this precision helps guide targeted interventions that can modify m^6^A patterns to achieve desired phenotypes, paving the way for precision epitranscriptomic engineering in agriculture.

The emergence of CRISPR-based technologies that target RNA, particularly CRISPR/Cas13, has opened new avenues for precise manipulation of m^6^A modifications at the transcript level. Unlike conventional CRISPR/Cas9 systems that target DNA, CRISPR/Cas13 is designed to bind to RNA and can be engineered for site-specific modifications (Yang and Patel 2024). Indeed, a catalytically inactive Cas13 (dead Cas13 or dCas13) can be fused to an m^6^A-writer gene or eraser gene, allowing for the selective addition or removal of m^6^A marks on target transcripts (Wei et al. 2022). This strategy offers a reversible and dynamic means for controlling mRNA fate without altering the underlying genomic sequence. A recent study developed programmable m^6^A editors in cotton by combining CRISPR/dCas13(Rx)—a specific subtype of CRISPR/dCas13 (VI-D)—with either the methyltransferase GhMTA or the demethylase GhALKBH10. These tools efficiently modulate m^6^A levels on *Calcium-transporting ATPase 1* (*GhECA1*) and *Drought-inducible 19* (*GhDi19*) transcripts within a 0- to 46-nucleotide window: the Targeted RNA Demethylation Editor (TDE) lowered m^6^A levels by 24–76%, whereas the Targeted RNA Methylation Editor (TME) raised them by 37–151%. Such precise modulation of m^6^A deposition significantly affected the phenotypes of cotton plants, notably enhancing root length and drought tolerance (Yu et al. 2024). Although CRISPR/Cas13-mediated m^6^A site editing is efficient, off-target effects remain a concern. In the future, it may be feasible to enhance the accuracy of m^6^A site recognition by utilizing additional or different reader domains in combination with dCas13-eraser or dCas13-writer fusion proteins.

RNA-based, CRISPR-free targeting systems can employ guide RNAs, such as linear or circular antisense RNAs, small nucleolar RNAs (snoRNAs), or ribozymes, to bind to specific RNA sequences via complementary pairing and recruit endogenous RNA modification enzymes (Gu et al. 2020; Song et al. 2024). For example, RESTART (RNA editing to specific transcripts for pseudouridine-mediated PTC readthrough) is a programmable RNA base editor that converts uridine to pseudouridine at stop codons, thereby suppressing premature termination codons (PTCs) in mammalian cells. RESTART utilizes an engineered guide snoRNA (gsnoRNA) in conjunction with the endogenous H/ACA snoRNP machinery to achieve precise pseudouridylation. Optimization of gsnoRNA scaffolds, along with a minor isoform of Dyskerin Pseudouridine Synthase 1 (DKC1) lacking a C-terminal nuclear localization signal, significantly enhanced PTC readthrough efficiency; RESTART induced robust pseudouridylation in primary cells with minimal off-target effects, preserving coding integrity and expression levels (Song et al. 2023a). In parallel, RNA base editing can exploit modification enzymes such as Adenosine deaminase acting on RNA 1 (ADAR1) and ADAR2, which catalyze adenosine-to-inosine (A-to-I) conversions, inosine being interpreted as guanosine by the translation machinery (Booth et al. 2023). Several studies have demonstrated that guide RNA–directed ADAR systems can effectively mediate targeted base conversions, underscoring the feasibility of programmable RNA editing (Song et al. 2024).

Integrating these RNA-editing approaches into the regulation of m^6^A marks holds significant promise for fine-tuning gene expression underlying key agronomic traits such as fruit ripening, flowering, and stress responsiveness. Although this field is still emerging, preliminary studies and theoretical models strongly support the potential of RNA editing for m^6^A-modified transcripts as a powerful tool for crop trait optimization. As these technologies mature, both CRISPR/Cas13-based and CRISPR-free RNA-editing platforms are expected to become indispensable methods for precision epitranscriptomic engineering in crop improvement.

### Small molecule-mediated m^6^A regulation

Small molecule inhibitors offer a complementary strategy to genetic and RNA-based interventions for modulating m^6^A modifications. These compounds are designed to target specific components of the m^6^A regulatory machinery and precisely control m^6^A levels. Indeed, small-molecule inhibitors can effectively modulate m^6^A levels by interfering with the catalytic activity of methyltransferases or demethylases (Deng et al. 2023). For example, a series of inhibitors targeting FTO have shown promising therapeutic activity in various cancer models. In a structure-based virtual screening study, rhein, a natural anthraquinone from medicinal herbs, was identified as an FTO inhibitor; rhein raises overall cellular m^6^A levels in mRNA, although its selectivity for FTO over ALKBH5 is limited (Chen et al. 2012). Analogs of α-ketoglutarate (α-KG) represent another class of FTO inhibitors with potential application as anti-tumor drugs; these compounds compete with α-KG by chelating iron within the FTO active site, thereby inhibiting its catalytic function (Su et al. 2018). In addition, inhibitors of m^6^A writers have been developed; for instance, the compound STM2457 is a potent and selective inhibitor of METTL3 that binds within the S-adenosyl methionine (SAM)-binding site of the METTL3–METTL14 complex, although it is not structurally related to SAM (Yankova et al. 2021). Moreover, small-molecule inhibitors targeting m^6^A readers have also been identified, such as CWI1-2, which selectively disrupts the binding of the RNA-binding protein IGF2BP2 (Insulin-like growth factor 2 mRNA-binding protein 2) to its m^6^A-modified target RNAs (Weng et al. 2022).

Collectively, these findings illustrate the versatility of small-molecule inhibitors in fine-tuning the m^6^A regulatory network. Additional small molecules inhibiting m^6^A may be identified and selected based on their capacity to modulate the liquid–liquid phase separation (LLPS) properties of m^6^A components and regulate their post-translational modifications (Jiang 2024; Shen 2024). However, no studies have yet elucidated how these small molecules control m^6^A modifications in plants, or how they affect plant growth, development, and environmental adaptation. The prospect of using small molecules to transiently alter m^6^A dynamics is particularly attractive, as it offers a non-genetic means of controlling gene expression with potential applications in crop stress management and quality improvement. Further research in this area, especially the discovery of efficient and broadly applicable small molecules, will be instrumental for improving crop yield and quality in agriculture.

### m^6^A-driven multi-omics integration with machine learning (ML)

Despite the identification of numerous m^6^A-related components, the mechanisms governing their site-specific regulation across different conditions remain only partially understood. Given the critical roles of the m^6^A modification in plant development and stress responses, integrating multi-omics datasets including genomics, transcriptomics, epigenomics, and proteomics with advanced machine learning techniques presents a promising strategy for unraveling these complexities. By combining condition-dependent m^6^A profiles, gene expression and genome-wide association studies (GWAS), researchers can pinpoint regulatory elements and signaling pathways modulated by m^6^A. Moreover, leveraging high-throughput sequencing technologies such as m^6^A-SAC-seq, ribosome profiling, and RNA decay sequencing, alongside machine learning algorithms, can facilitate the construction of comprehensive epigenetic regulatory networks. Compared with using transcriptome deep sequencing (RNA-seq) or ribosome profiling (Ribo-seq) data alone, integrative models that combined both types of data significantly enhanced the prediction of flowering-related genes in maize, leading to the identification of 20 candidate genes, 18 of which had not been previously reported to influence flowering (Han et al. 2023). When using genomic data from the 1,001 Genomes Project for Arabidopsis accessions (Alonso-Blanco et al. 2016) alongside single-omics datasets such as RNA-seq and DNA methylome (Kawakatsu et al. 2016) to predict flowering-related genes in Arabidopsis, the overlap among important gene sets was low, suggesting that each omics layer provides complementary information (Wang et al. 2024b). Notably, Random Forest models integrating genomic and transcriptomic data with methylome data achieved the best predictive performance, surpassing models based on any single-omics dataset. These findings demonstrate that integrating multi-omics data can substantially enhance the predictive power of machine learning approaches. Future research should therefore incorporate RNA modification profiling (e.g., CAM-seq) in Arabidopsis, in combination with the genomic data from the 1,001 Genomes Project, to predict RNA methylation patterns and phenotypic variation. Furthermore, the inclusion of emerging RNA degradation datasets will increase the complexity and depth of the multi-omics network, ultimately improving the robustness and reliability of predictive models. This integrative framework should enhance our understanding of m^6^A-mediated regulation of gene expression and drive precision strategies for crop improvement.

## Conclusion

Accumulating evidence underscores the pivotal role of the m^6^A modification in crop quality and yield. Through orchestrating key post-transcriptional steps such as mRNA splicing, stability, and translation, m^6^A serves as a dynamic regulatory hub that enables plants to fine-tune gene expression in response to developmental cues and environmental challenges (Fig. 1). This regulation directly influences critical agronomic traits including flowering time, fruit ripening, stress resilience, and ultimately, overall crop productivity (Figs. 2 and 3).

Despite these advances, several limitations remain in current m^6^A research, particularly in research on economically important crops outside of well-studied model systems like Arabidopsis. Although large-scale phylogenetic analyses have provided insights into their origin and evolution (Zhang et al. 2024a), a systematic identification and functional profiling of m^6^A regulatory components in a broader range of crops is lacking. While the core machinery has been characterized in model plants, the incomplete profiling in many crop species hinders our ability to translate these fundamental insights into practical applications for crop improvement.

Moreover, the mechanisms underlying the specificity of RNA m^6^A modifications in plants require further elucidation. Although m^6^A is predominantly deposited within conserved motifs such as RRACH (He and He 2021; Roundtree et al. 2017; Shen 2023; Tang et al. 2023), the precise determinants that guide this modification to specific sites along the transcript require further studies. Earlier research has demonstrated that m^6^A is predominantly enriched in the last exon and in long internal exons. This distribution is further influenced by the underlying exon architecture and regulated by the Exon Junction Complex (EJC) in mammals (He et al. 2023b). However, recent findings suggest that the active deposition of m^6^A in plants may be governed by specific genomic features near the stop codon rather than by the EJC (Wang et al. 2024a). These differences in m^6^A modification mechanisms between the plant and mammalian kingdoms suggest the existence of previously uncharacterized regulatory programs for m^6^A deposition on mRNA, which should be elucidated. Moreover, m^6^A levels vary across different tissues within the same species (Wang et al. 2024a). This variation may be attributed to the tissue-specific accumulation of certain proteins involved in m^6^A modification. Key questions also include how accessory proteins and contextual factors contribute to the spatial and temporal control of m^6^A deposition and how the dynamic changes of m^6^A levels differ among various conditions, such as in plant responses to abiotic and biotic stresses. One possibility is that individual stress conditions can activate specific, environment-dependent factors to elicit the selective modification of target RNAs. For example, the m^6^A reader ECT8 forms phase-separated condensates in the cytoplasm and selectively recruits m^6^A-modified mRNAs, such as those for the ABA receptor gene *PYRABACTIN RESISTANCE 1-LIKE 7* (*PYL7*), into stress granules to restrict their translation in response to ABA (Wu et al. 2024). In addition, histone modifications may also be associated with the m^6^A modification, as about 70% of m^6^A-modified sites in RNA overlap with H3K36me3 sites in the corresponding genomic DNA of mammals (Huang et al. 2019). In plants, the H3K36me2 chromatin mark is highly associated with m^6^A modifications of the corresponding RNAs (Shim et al. 2020). These findings suggest a role for histone modifications in RNA m^6^A deposition, although the exact mechanism is unclear. Thus, resolving these questions will be essential for harnessing m^6^A modifications to achieve targeted gene regulation in Arabidopsis and crops.

The functional consequences of m^6^A modifications on mRNA fate are complex and appear to be highly context-dependent. It remains unclear how the precise locations of m^6^A marks on mRNA influence transcript stability, miRNA biogenesis, translation efficiency, and ultimately protein production in plants (Amara et al. 2022; Bhat et al. 2020; Cai et al. 2024a; Chen et al. 2024; Li et al. 2024; Prall et al. 2023; Shoaib et al. 2021; Tang et al. 2021; Wei et al. 2024; Zhang et al. 2022b; Zhong et al. 2024). For instance, m^6^A deposition within the coding sequence tends to stabilize mRNAs involved in strawberry ripening, whereas the presence of modifications in the 3′ UTR or near the stop codon are negatively associated with mRNA abundance during tomato ripening (Zhou et al. 2019, 2021). Although numerous studies suggest that the m^6^A modification primarily stabilizes RNA, thereby modulating development, circadian rhythms, and stress responses (Anderson et al. 2018; Wang et al. 2021; Wei et al. 2018), there is also evidence indicating that m^6^A promotes RNA degradation during stem cell fate determination in shoots and during hypocotyl growth (Shen et al. 2016; Yang et al. 2023). A possible explanation for this discrepancy is that different m^6^A-binding protein complexes, in conjunction with specific initiation factors, elongation factors, or 3′ processing factors, may be selectively recruited to distinct mRNA regions, thus influencing RNA metabolism during specific developmental stages or environmental responses. Addressing these issues is critical for understanding how differential m^6^A deposition can be leveraged to modulate specific agronomic traits, such as enhancing fruit quality or improving stress tolerance.

Advances in high-throughput quantitative profiling and sequencing, RNA-editing technologies, and computational modeling hold great promise for overcoming current limitations in m^6^A research (Fig. 4). As our ability to accurately map and manipulate m^6^A modifications improves, it will become increasingly feasible to design targeted interventions that fine-tune gene expression with precision. However, practical application of these technologies requires a careful consideration of potential challenges. For instance, CRISPR/Cas13-mediated RNA editing, while highly specific, may exhibit off-target effects, necessitating rigorous validation and optimization of guide RNAs and effector proteins. Furthermore, efficient and stable delivery systems of RNA-editing complexes and small-molecule inhibitors into crop plants are actively being developed. In addition, regulatory frameworks governing genetically edited crops and the approval framework for small-molecule interventions need to be addressed comprehensively to facilitate real-world agricultural deployment. Ultimately, addressing these technical, regulatory, and delivery-related challenges will pave the way for next-generation agricultural practices that enhance crop quality and yield while ensuring resilience in the face of environmental challenges. In conclusion, while m^6^A modifications have emerged as critical regulators of crop performance, further research is needed to fully exploit their potential in agricultural biotechnology. A deeper understanding of the regulatory networks, specificity determinants, and positional effects of m^6^A, coupled with practical strategies to overcome the associated biotechnological challenges, will provide the necessary foundation for innovative crop improvement strategies with significant implications for global food security and sustainable agriculture.

## Data Availability

Data sharing is not applicable to this article as no datasets were generated or analyzed during the current study.
